# Supplementary material for: MetaRibo-Seq measures translation in microbiomes
Source: Nat Commun. 2020 Jun 29;11:3268. doi: 10.1038/s41467-020-17081-z (PMC7324362; doi:10.1038/s41467-020-17081-z)
Supplement: Supplementary file 10 — Supplementary Data 7 [file 41467_2020_17081_MOESM10_ESM.zip › File2/Confidence_High_Taxonomy/335719_out.krona.html]

Javascript must be enabled to view this page.

members
magnitude
magnitudeUnassigned
count
unassigned
taxon
rank

335719\_out

3

3
2
superkingdom

phylum
976
3

class
200643
3

order
171549
3

171552
3
family

genus
838
3

2292365
3

SRS015794\_contig\_number\_contig-100\_847.87326SRS023526\_contig\_number\_12275SRS077849\_contig\_number\_30649
species
